# Vitamin D receptor gene associations with pulmonary tuberculosis in a Tibetan Chinese population

**DOI:** 10.1186/s12879-016-1699-4

**Published:** 2016-09-05

**Authors:** Qunying Hu, Zhengshuai Chen, Guinian Liang, Fangping Mo, Hengxun Zhang, Shilin Xu, Yuhe Wang, Longli Kang, Tianbo Jin

**Affiliations:** 1 Key Laboratory for Basic Life Science Research of Tibet Autonomous Region, School of Medicine, Xizang Minzu University, Xianyang, Shaanxi 712082 China; 2 Key Laboratory for Molecular Genetic Mechanisms and Intervention Research on High Altitude Disease of Tibet Autonomous Region, School of Medicine, Xizang Minzu University, Xianyang, Shaanxi 712082 China; 3 Key Laboratory of High Altitude Environment and Genes Related to Diseases of Tibet Autonomous Region, School of Medicine, Xizang Minzu University, Xianyang, Shaanxi 712082 China; 4 School of Life Sciences, Northwest University, Xi’an, Shaanxi 710069 China; 5 National Engineering Research Center for Miniaturized Detection Systems, Xi’an, Shaanxi 710069 China; 6 Affiliated Hospital of Xizang Minzu University, Xianyang, Shaanxi 712082 China

**Keywords:** *VDR*, SNP, Tibetan Chinese population, PTB

## Abstract

**Background:**

The vitamin D receptor (*VDR*) mediates the immunological function of vitamin D3, which activates macrophages, and vitamin D deficiency has been linked to tuberculosis risk. Single nucleotide polymorphisms (SNPs) in *VDR* may influence the function of vitamin D and susceptibility to tuberculosis.

**Methods:**

This study included 217 patients with pulmonary tuberculosis (PTB) and 383 healthy subjects in a Tibetan Chinese population living in and near Xi’an. Association analyses of SNPs in *VDR* were performed with the SPSS 17.0 statistical packages, SNP stats software, Haploview software package (version 4.2), and the SHEsis software platform.

**Results:**

Our results revealed a correlation between three SNPs (rs11574143, odds ratio [OR]: 1.47, 95 % confidence interval [CI]: 1.11 - 1.94, *p* = 0.006, *p*-adjust = 0.030; rs11574079, OR: 0.48, 95 % CI: 0.25 - 0.92, *p* = 0.023, *p*-adjust = 0.115; rs11168287, OR: 2.55, 95 % CI: 2.00 - 3.25, *p* = 1.730E-14, *p*-adjust = 0.865E-13) and PTB based on Chi-square tests. We observed the allele “A” of rs11574143 and rs11168287 increased the PTB risk and the allele “A” of rs11574079 provided a protective effect against PTB.

**Conclusions:**

The goal of this study was the identification of putative associations between five SNPs (rs11574143, rs7975232, rs11574079, rs3819545 and rs11168287) in *VDR* and susceptibility to PTB. Our findings demonstrated associations between *VDR* polymorphisms and PTB development.

## Background

Pulmonary tuberculosis (PTB), which is, caused by the human pathogen *Mycobacterium tuberculosis,* is a major cause of morbidity and mortality in human populations worldwide. Susceptibility to disease upon *M. tuberculosis* infection is influenced by the agent, environmental and host genetic factors [1]. Each year more than 9 million people are infected by PTB and more than 1.7 million succumb to PTB annually [2]. Cell-mediated immunity is essential for the suppression of an *M. tuberculosis* infection as it is an intracellular parasite [3]. The fact that only 10 % of those infected with *M. tuberculosis* progress to clinical disease revealed that genetic factors, as well as environmental factors are involved in the pathophysiology of PTB [4]. In addition, the host genetic basis of PTB has been confirmed by twin studies that indicated a two times higher risk of disease in identical twins compared to non-identical twins [5]. There is a much higher risk of severe disease and death in children than in adults, therefore, the pediatric tuberculosis remains a public health emergency [6]. Many studies have shown that genetic factors play important roles in PTB disease development, and vitamin D receptor (*VDR*) gene has recently been found to be interesting candidate genes.

The active form of vitamin D, 1-25-dihydroxyvitamin D3, is an important hormone that modulates the activity of different defense and immune cells, including lymphocytes, monocytes, macrophages and epithelial cells [7]. Vitamin D restricts *M. tuberculosis* growth in macrophages through the production of the anti-microbial peptide, cathelicidin [8].Many studies showed an association between vitamin D deficiency and certain diseases such as systemic lupus erythematosus [9], diabetes mellitus [10], tuberculosis [11], etc. During a tuberculosis infection, vitamin D binds to *VDR* in macrophages, and this binging activates synthesis of the antimicrobial peptide cathelicidin, which restricts *M. tuberculosis* intracellular growth in macrophages [12], and eliminates *Mycobacterium tuberculosis* in phagolysosomes [13]. Thus, polymorphisms in *VDR* may contribute to PTB susceptibility.

In this study, we evaluated the roles of *VDR* gene polymorphisms and haplotypes on PTB susceptibility by conducting an extensive association analysis of a case–control study in a Tibetan Chinese population. We found considerable evidence for associations between three SNPs and PTB susceptibility.

## Methods

### Study participants

From October 2012 to September 2013, we recruited 217 patients to participate in an ongoing molecular epidemiological study at the Department of Respiratory Physicians of the Tangdu Hospital affiliated with The Fourth Military Medical University in Xi’an, China. The inclusion criteria in the case group were newly diagnosed pulmonary tuberculosis patients, Tibetan ethnic, have symptoms of PTB, positive sputum smear and chest radiography consistent with active disease. The exclusion criteria in case group were HIV positive, known to present diabetes mellitus and other severe diseases, and consuming immunosuppressive drugs.

From June 2012 to July 2013, we also recruited a random sample of 383 unrelated healthy individuals at the medical center of Tangdu Hospital to serve as controls. All of the study participants were Tibetan Chinese living in Xi’an and nearby. Controls subjects had no history PTB and no evidence of prior PTB noted on chest radiography. We enlisted them without regard to their age, gender, or disease stage.

### SNP selection and genotyping

We selected five single nucleotide polymorphisms (SNPs) in *VDR* that had with minor allele frequencies (MAF) > 5 % and were associated with PTB in the HapMap Asian population. We extracted genomic DNA from peripheral blood samples using the GoldMag-Mini Whole Blood Genomic DNA Purification Kit (GoldMag Ltd. Xi'an, China) according to the manufacturer's protocol. Sequenom MassARRAY Assay Design 3.0 Software was used to design primers for amplification and extension reactions [14]. SNP genotyping using the standard protocol recommended by the manufacturer was performed by Sequenom MassARRAY RS1000 [14]. Finally, Sequenom Typer 4.0 Software was used to perform the data management and analysis [14, 15].

### Statistical analysis

We used Microsoft Excel and the SPSS 17.0 statistical package (SPSS, Chicago, IL) to perform statistical analyses. All of the p-values presented in this study are two-sided, and p ≤ 0.05 was used as the threshold of statistical significance. Departure from Hardy-Weinberg Equilibrium (HWE) of each SNP frequency was assessed by an exact test on the control subjects. We calculated the genotype frequencies between the cases groups and control groups by Chi-square test [16].

Odds ratios (OR) and 95 % confidence intervals (CI) were calculated by unconditional logistic regression analyses [17]. We used the web-based software SNP stats to test the associations between SNPs and the risk for PTB in four genetic models (codominant, dominant, recessive, and additive) [18].

The software platform (http://sampsize.sourceforge.net/iface/s3.html) was used for evaluating the statistical power of this case-control study. We used the Haploview software package (version 4.2) and SHEsis software platform (http://www.nhgg.org/analysis/) for analyses of linkage disequilibrium, haplotype construction, and genetic association at polymorphism loci [19, 20].

## Results

The PCR primers for the 5 selected SNPs, which were designed using the Sequenom MassARRAY Assay Design 3.0 Software, were listed in Table 1.

**Table 1:** Primers used in this study

SNP ID	1st-PCR primer sequence	2^nd^-PCR primer sequence	UEP sequence
rs11574143	ACGTTGGATGAACTGTGTCTGCCATTAGAG	ACGTTGGATGTAGGAGTCCTGTTTCTGCAC	cataCTCCAGGTCACTGGCA
rs7975232	ACGTTGGATGTGCCGTTGAGTGTCTGTGTG	ACGTTGGATGTAGAGAAGAAGGCACAGGAG	taagGGAGCTCTCAGCTGGGC
rs11574079	ACGTTGGATGTGAGGAAGAGCCTATGCTGG	ACGTTGGATGGAGGGAAGCACCATCTCTTG	cCCATCTCTTGCAGTAGC
rs3819545	ACGTTGGATGTTATCCTGTGGGTAGATCGG	ACGTTGGATGTCATGGTCATTTAGGTTCGG	AGGTTCGGTCTTTGGCT
rs11168287	ACGTTGGATGGGAGGAAAAGTCCAGATTTG	ACGTTGGATGGGGTGAGGAGAAATGAGTTG	gggaTTGTTCTAATCTCTCTCCTAACA

*SNP* single nucleotide polymorphism; *PCR* polymerase chain reaction; *UEP* unique base extension primer

Basic information of candidate SNPs in this study was shown in Table 2. We also adjusted the *p*-value by Bonferroni correction. It listed the minor allele frequency (MAF) of cases and controls, we found there existed a correlation between three loci (rs11574143, OR: 1.47, 95 % CI: 1.11 - 1.94, *p* = 0.006, *p*-adjust = 0.030; rs11574079, OR: 0.48, 95 % CI: 0.25 - 0.92, *p* = 0.023, *p*-adjust = 0.115; rs11168287, OR: 2.55, 95 % CI: 2.00 - 3.25; *p* = 1.730E-14, *p*-adjust = 0.865 E-13) and PTB based on Chi-square tests. Rs7975232 and rs3819545 had no connection with PTB susceptibility. All of the tested SNPs conformed to HWE in the control group of this study (*p* > 0.05). Post-hoc power value we calculated of rs11574143, rs7975232, rs11574079, rs3819545, rs11168287 were 77.59 %, 45.41 %, 62.96 %, 4.13 %, 100 %, respectively.

**Table 2:** Basic information of candidate SNPs in this study

SNP-ID	Allele	Gene	Loc.	MAF		HWE-*p*	OR (95 % CI)	*p*-value	*p*-adjusted
				Case	Control				
rs11574143	A/G	VDR	12q13.11	0.265	0.197	1	1.47 (1.11-1.94)	0.006*	0.030*
rs7975232	A/C	VDR	12q13.11	0.327	0.277	0.799	1.27 (0.98-1.64)	0.066	0.330
rs11574079	A/G	VDR	12q13.11	0.028	0.056	1	0.48 (0.25-0.92)	0.023*	0.115
rs3819545	T/C	VDR	12q13.11	0.274	0.279	0.799	0.97 (0.75-1.27)	0.847	1
rs11168287	A/G	VDR	12q13.11	0.569	0.341	0.569	2.55 (2.00-3.25)	1.730E-14*	0.865E-13*

*SNP* single nucleotide polymorphism; *MAF* minor allele frequency; *HWE* Hardy-Weinberg Equilibrium; *OR* odds ratio; *CI* confidence interval

*p-value <0.05 indicates statistical significance

The relationships between rs11574143, rs11574079, rs11168287 and PTB risk were listed in Table 3. We observed the allele “A” of rs11574143 and rs11168287 increased the PTB risk and the allele “A” of rs11574079 provided a protective effect against PTB.

**Table 3:** Associations between the SNP genotypes of VDR and the risk of PTB

SNP-ID	Model	Genotype	Control (N,%)	Case (N,%)	OR (95 % CI)	*p*-value	*p*-adjusted
		G/G	246 (64.6 %)	116 (53.5 %)	1		
	Codominant	A/G	120 (31.5 %)	87 (40.1 %)	1.54 (1.08-2.19)	0.024*	0.12
		A/A	15 (3.9 %)	14 (6.5 %)	1.98 (0.92-4.24)		
rs11574143	Dominant	G/G	246 (64.6 %)	116 (53.5 %)	1		
		G/A-A/A	135 (35.4 %)	101 (46.5 %)	1.59 (1.13-2.23)	0.0077*	0.039*
	Recessive	G/G-G/A	366 (96.1 %)	203 (93.5 %)	1		
		A/A	15 (3.9 %)	14 (6.5 %)	1.68 (0.80-3.56)	0.18	0.90
	Log-additive	---	---	---	1.48 (1.11-1.96)	0.0066*	0.033*
		G/G	341 (89 %)	205 (94.5 %)	1		
	Codominant	A/G	41 (10.7 %)	12 (5.5 %)	0.49 (0.25-0.95)	0.053	0.265
		A/A	1 (0.3 %)	0 (0 %)	0.00 (0.00-NA)		
rs11574079	Dominant	G/G	341 (89 %)	205 (94.5 %)	1		
		A/G-A/A	42 (11 %)	12 (5.5 %)	0.48 (0.24-0.92)	0.021*	0.105
	Recessive	G/G-A/G	382 (99.7 %)	217 (100 %)	1		
		A/A	1 (0.3 %)	0 (0 %)	0.00 (0.00-NA)	0.34	1
	Log-additive	---	---	---	0.47 (0.25-0.91)	0.018*	0.09
		G/G	168 (44.1 %)	43 (19.8 %)	1		
	Codominant	A/G	166 (43.6 %)	101 (46.5 %)	2.38 (1.57-3.60)	<0.0001*	<0.0005*
		A/A	47 (12.3 %)	73 (33.6 %)	6.07 (3.69-9.97)		
rs11168287	Dominant	G/G	168 (44.1 %)	43 (19.8 %)	1		
		A/G-A/A	213 (55.9 %)	174 (80.2 %)	3.19 (2.16-4.72)	<0.0001*	<0.0005*
	Recessive	G/G-A/G	334 (87.7 %)	144 (66.4 %)	1		
		A/A	47 (12.3 %)	73 (33.6 %)	3.60 (2.38-5.46)	<0.0001*	<0.0005*
	Log-additive	---	---	---	2.46 (1.92-3.15)	<0.0001*	<0.0005*

*OR* odds ratio; *CI* confidence interval; *: p-value <0.05 indicates statistical significance. *SNP* single nucleotide polymorphism; *VDR* vitamin D receptor; *PTB* pulmonary tuberculosis

For rs11574143, In the codominant model, the genotype “A/G” (OR =1.54; 95 % CI, 1.08 - 2.19; *p* = 0.024, *p*-adjust = 0.12) increased PTB risk by 1.54-fold; In the dominant model, the genotype “G/A” and “A/A” (OR = 1.59; 95 % CI, 1.13 - 2.23; *p* = 0.0077, *p*-adjust = 0.039) increased PTB risk by 1.59-flod; In the additive model, the allele “A” increased PTB risk by 1.48-fold (OR = 1.48; 95 % CI, 1.11 - 1.96; *p* = 0.0066, *p*-adjust = 0.033). We also observed another susceptibility SNP, rs11168287, *p*-value < 0.0001 (*p*-adjust < 0.0005) for all of the four models in SNP analyses. In the codominant model, the genotype “A/G” (OR = 2.38; 95 % CI, 1.57 - 3.60) and “A/A” (OR = 6.07; 95 % CI, 3.69 - 9.97) increased PTB risk by 2.38-fold and 6.07-fold respectively; In the dominant model, the genotype “A/G” and “A/A” increased more than 3-fold PTB risk (OR = 3.19; 95 % CI, 2.16 - 4.72); In the recessive model, the genotype “A/A” increased PTB risk by 3.60-fold (OR = 3.60; 95 % CI, 2.38 - 5.46). In the additive model, the allele “A” increased PTB risk by 2.46-fold (OR = 2.46; 95 % CI, 1.92 - 3.15).

The SNP rs11574079 correlated with a protective effect against PTB in the dominant model (OR: 0.48, 95 % CI: 0.24 - 0.92, *p* = 0.021, *p*-adjust =0.105) and in the additive model (OR: 0.47, 95 % CI: 0.25 - 0.91, *p* = 0.018, *p*-adjust = 0.09).

**Fig. 1:**
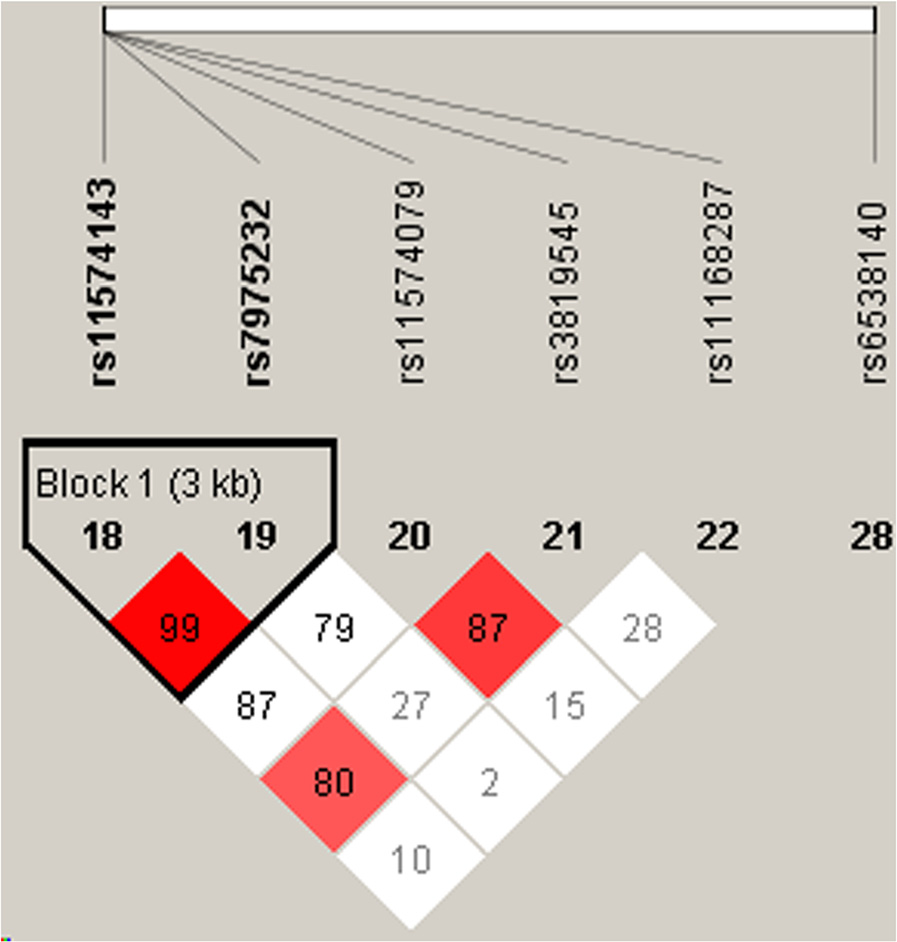
Linkage disequilibrium patterns of five SNPs in VDR

A block (rs4947492 and rs12718945) was detected in studied *VDR* SNPs by haplotype analyses (Fig. 1). The results of the association between the *VDR* haplotype and the risk of PTB were listed in Table 4. Altogether there were three haplotypes and only the haplotype “A/A” was found to increase risk of suffering from PTB by 1.45-fold (OR = 1.45; 95 % CI, 1.09 - 1.94; *p* = 0.01). We adjusted the *p*-value by Bonferroni correction, the haplotype “A/A” still existed a statistically significant difference (*p* = 0.02).

**Table 4:** Haplotype analysis results of rs11574143 and rs7975232 in VDR

SNP ID	rs11574143	rs7975232	Freq	OR (95 % CI)	*p*-value	*p*-adjusted
Haplotype	G	C	0.704	1	---	
	A	A	0.221	1.45 (1.09-1.94)	0.01*	0.02*
	G	A	0.074	0.84 (0.52-1.36)	0.49	0.98

VDR: vitamin D receptor; SNP: single nucleotide polymorphism; OR: odds ratio; CI: confidence interval

*p-value <0.05 indicates statistical significance

## Discussion

Our comprehensive analysis of SNPs in *VDR* suggests that *VDR* genotypes and haplotypes associate with PTB risk. We found two PTB risk associated SNPs, rs11574143 and rs11168287, and one PTB protective SNP, rs11574079, in *VDR*. The other two *VDR* SNPs, rs7975232 and rs3819545, had did not associate with PTB susceptibility.

In previously published studies, there is no research reported that the association between *VDR* gene and PTB susceptibility. We for the first time studied the correlations between three SNPs (rs11574143, rs11168287 and rs11574079) in *VDR* gene and PTB susceptibility in a Tibetan Chinese population, rs3819545 showed no correlations with PTB susceptibility, and we got some valuable results. Further studies should involve some others regions and ethnic groups.

A few studies have analyzed the association between the *VDR* ApaI (rs7975232) polymorphism and TB susceptibility. A study conducted by Selvaraj et.al. in the Indian population shows that the ‘A/A’ genotype is associated with resistance to pulmonary TB in males, but not in females [21]. In a study carried out in pulmonary TB patients of south India, the Bb genotype of *VDR* BsmI was associated with susceptibility to TB, whereas A/A genotype of ApaI and B/B genotype of BsmI were associated with resistance to pulmonary TB [22, 23]. Our results are inconsistent with the results of some previously published studies, a possible explanation could be their ethnic differences.

Comparisons with other researches showed that population or various ethnicities give different results. Many studies have suggested associations between *VDR* SNPs and PTB in variety populations [22, 24, 25]. The different results shown by Central Indian and South Indian populations may be due to the diversity of these two Indian populations and their difference in origins [26]. A latest study was in accordance with those obtained by other authors for West African populations, but in total discrepancy with the results for Indian, Iranian and Chinese populations [27]. Several case-control studies, performed in populations with high TB incidence, had studied the correlation between the *VDR* gene polymorphisms and genetic susceptibility to PTB, but the results were inconclusive, presumably because the populations were ethnically and geographically different and genetically diverse [25, 28, 29]. Wei et al. reported no association was found between *VDR* polymorphisms (either allele or genotype) and susceptibility to pediatric PTB in the Chinese Han population [30]. Also, a study in Cambodia [31] and Tanzania [32] found no associations between *VDR* gene and PTB. These differing results could be caused by gene-environment interaction, gene-gene interaction, and gene-agent interactions. These reports suggest that the differing association results between *VDR* genotypes in different ethnic populations may result from gene-environment [33] and gene-gene interactions [34, 35].

There are other factors that may influence the results. It is known that before vitamin D enters the macrophage, inactive vitamin D in serum could be bound to vitamin D binding protein or in a free state*.* A recent study showed that VDR variant (rs7968585) can predict risk for hip fracture, cancer, myocardial infarction, and mortality among older Caucasian individuals with a low vitamin D status [36]. This association was only apparent in individuals with a low vitamin D status, highlighting the importance of taking into account gene-environment interactions. Vitamin D status appears to be involved in the activation of macrophages and restricts the intracellular development of mycobacteria [37]. This effect of vitamin D may be influenced by polymorphisms in the *VDR* gene. However, although the relationship between the internal environment and the host gene is unknown, it may explain the heterogeneity observed in the association studies and it should be addressed as an influencing factor. The polymorphic variants of *VDR* gene along with other gene and environmental factors may be responsible for an altered cell-mediated immunity against *M. tuberculosis* in a susceptible or resistant host. Although several investigators reported and suggested that the *VDR* polymorphism may be of immune-regulatory significance for PTB but it is not clear that the polymorphisms determine susceptibility to the development of clinical disease or susceptibility to infection. Further studies are required to determine how VDR polymorphisms influence susceptibility to infection or to clinical disease development. In order to understand the pathogenesis of pulmonary TB that is useful for prophylaxis and therapy. Ethnic-specific genetic association with TB susceptibility may guide TB therapy and prophylaxis in an ethnic-specific manner. A post hoc power analysis was conducted and the power analysis showed that except rs11168287, other SNPs failed to reach minimum level (80 %) for this case-control study. In this case, we could not conclude that the difference was not statistically significant, but need to increase sample size to verify. Future research should pay attention to this point.

Because we limited ethnicity of our subjects to Tibetan Chinese, and limited the locations of our subjects to Xi’an City and its surrounding area, there were no substantial population admixtures in our study populations. However, some limitations should be considered when interpreting our results. Our sample size was small and only included 217 PTB cases. Also, gene-environment and gene-gene interactions may effect *VDR* expression and activity. And the assessment of the environmental factors such as tobacco, diet and alcohol consumption and the possibility of inaccurate exposure should be considered. The susceptibility and resistance to PTB are the result of the interactions between environment, socioeconomic status and host genes, therefore, further studies are required to elucidate the complex associations between the *VDR* gene variants and the immune response against PTB. These data will lead to a better understanding of the immunological and genetic pathways in tuberculosis, and will provide new ideas for potential treatments and prophylaxes to reduce the prevalence and incidence of PTB caused by *M. tuberculosis* infections.

## Conclusions

Our findings demonstrated that genetic variants of *VDR* play complex roles in the development of PTB and provided new evidence for associations between SNPs and haplotypes of *VDR* and the risk of PTB. This study offers new information on the relationship between *VDR* polymorphisms and PTB .Moreover, this study reveals the molecular markers associated with of PTB susceptibility and could therefore be used as diagnostic and prognostic markers for PTB patients in clinical study.

## Abbreviations

*VDR*, vitamin D receptor; SNP, single nucleotide polymorphism; PTB, pulmonary tuberculosis; MAF, minor allele frequencies; HWE, Hardy-Weinberg Equilibrium; OR, odds ratio; CI, confidence interval
